# Distribution, abundance and ecological associations among two species of native mussels (*Gonidea angulata* and *Margaritifera falcata*) and one invasive clam species (*Corbicula fluminea*) in the Columbia River Basin, North America

**DOI:** 10.1007/s00027-026-01314-2

**Published:** 2026-05-21

**Authors:** Nathaniel G. Neal, Stephen M. Bollens, Alexa N. Maine, Timothy D. Counihan, Gretchen Rollwagen-Bollens

**Affiliations:** 1 School of the Environment, Washington State University, Vancouver, WA USA; 2 Confederated Tribes of the Umatilla Indian Reservation, Freshwater Mussel Research and Restoration Project, Walla Walla, Washington, USA; 3 Western Fisheries Research Center, Columbia River Research Laboratory, Cook, WA USA; 4 Department of Ecology and Evolutionary Biology, University of Colorado, Boulder, CO 80309 USA

**Keywords:** Freshwater mussels, Freshwater clams, Aquatic invasive species, Habitat associations

## Abstract

Native freshwater mussels are declining globally, thus, determining factors associated with bivalve distribution and abundance is critical for native mussel conservation. In the Columbia River Basin, *Gonidea angulata* (western ridged mussel) is highly threatened, while *Margaritifera falcata* (western pearlshell) and the invasive clam *Corbicula fluminea* (Asian clam) are more common. We conducted 147 snorkel surveys, collected environmental data and performed statistical tests to quantify which variables were significantly associated with presence and abundance of these taxa. Variation in *G. angulata* presence and/or abundance were best explained by impervious surface proportion and annual air temperatures (mean and maximum). *Margaritifera falcata* presence and/or abundance were best explained by host fish presence, *G. angulata* presence, dissolved oxygen, and elevation. *Corbicula fluminea* presence and/or abundance were best explained by substrate size, impervious surface proportion, and annual air temperatures. Microhabitat and landscape variables explained some variation in bivalve presence and abundance, but the inclusion of watershed as a random effect increased the explanatory value. Consideration of species-specific habitat preferences and watershed-level variation could improve regional management in response to native mussel declines and invasive clam expansion.

**Supplementary Information:**

The online version contains supplementary material available at 10.1007/s00027-026-01314-2.

## Introduction

Native freshwater mussels are a highly imperiled faunal group worldwide, including in North America where populations have declined substantially in the past 100 years (Bogan 1993; Ferreira-Rodríguez et al. 2019). Over 65% of freshwater bivalve taxa in North America are classified as endangered, threatened, or vulnerable, with the remaining species susceptible to further declines due to population fragmentation (Haag 2012; Haag and Williams 2014). Extrinsic contributors to such declines include invasive species, anthropogenic disturbances, and changing climate conditions (Sousa et al. 2008; Ferreira-Rodríguez et al. 2019). Such threats may be exacerbated by intrinsic limitations of native freshwater mussels, which can include slow growth, long lifespans, and reliance on host fish for dispersal of a short-term parasitic larval (glochidia) phase (Haag 2012; Ferreira-Rodríguez et al. 2019).

Native freshwater mussels are important to aquatic systems due to their high capacity to filter water, their contributions to nutrient cycling, and their redistribution of particles from pelagic to benthic habitats (Vaughn and Hakenkamp 2001; Vaughn et al. 2008; Haag 2012). Thus, the presence of mussel beds and their biomass and assemblage composition can markedly structure habitats and modify food webs (Vaughn 2018), making declines in mussel populations of particular concern.

The Columbia River Basin (CRB), USA, is located on the west coast of North America with a largely snowmelt-fed drainage area of 567,000 km^2^ that extends across parts of seven USA states and two Canadian provinces (Matheussen et al. 2000). The CRB is inhabited by at least eight species of native freshwater mussels (Chong et al. 2008; Williams et al. 2017), including the western ridged mussel (*Gonidea angulata*) (Lea, 1838), the western pearlshell (*Margaritifera falcata*) (Gould, 1850), and multiple species of “floater” mussels (*Anodonta* spp.) (Linnaeus, 1758). Of interest to this study, *G. angulata* is highly threatened and a petition for its federal protection was submitted in 2020 (Blevins et al. 2017, 2020), while *M. falcata* is a more common and widespread native species, yet it is still under threat (International Union for Conservation of Nature 2016; Blevins et al. 2017). These two native mussels occasionally co-occur and their historical ranges significantly overlap (Blevins et al. 2017). Comparison of the distribution, abundance, and ecological associations (i.e., interactions between and among organisms and their environment) of these two native bivalves could provide insight into species-specific management strategies.

Freshwater mussel species vary in their substrate preference, temperature tolerance, and host fish specificity (Vannote and Minshall 1982; Holland-Bartels 1990; Valeria et al. 2025). Broad similarities shared between native mussel species include positive associations with hydrologically stable systems (Strayer 1993; Brown et al. 2010; Hegeman et al. 2014) and negative associations with anthropogenic disturbances such as construction, urbanization, and land use change resulting in sedimentation or habitat instability (Brim Box and Mossa 1999; Brown et al. 2010; Gillis et al. 2017). Impervious surfaces such as roads or other anthropogenic developments can increase run-off and result in higher flow variability following precipitation events (Smith and Smith 2015), limiting habitat suitability for freshwater mussels in proximate streams (Bukaveckas et al. 2023). Nutrient runoff and sediment erosion can negatively affect both freshwater mussels (Brim Box and Mossa 1999) and host fish (Zaimes et al. 2019).

Considering individual species, *G. angulata* abundance has been found to be positively correlated with greater substrate embeddedness and higher proportions of sand and gravel substrate (Vannote and Minshall 1982; Stanton et al. 2012; Davis et al. 2013; Snook et al. 2019). Other ecological associations of *G. angulata* include positive associations with higher dissolved oxygen concentrations (Snook et al. 2019) and increased availability of flow refuges (Vannote and Minshall 1982; Davis et al. 2013). Multiple species of sculpin, including *Cottus marginatus* (Bean, 1881), *Cottus pitensis* (Bailey and Bond, 1963), and *Cottus confusus* (Bailey and Bond, 1963), are confirmed host fish for *G. angulata* glochidia (Spring Rivers 2007; Stanton et al. 2012; O’Brien et al. 2013). In comparison, *M. falcata* rely on salmonids as host fish species (Murphy 1942; O’Brien et al. 2013) and *M. falcata* populations have been documented to be positively correlated with salmonid presence in coastal watersheds (Scully-Engelmeyer et al. 2023). Additionally, *M. falcata* exhibit preferences for a greater proportional composition of gravel and occasional boulders that allow for improved oxygen circulation (Vannote and Minshall 1982) and indeed, mussel density was positively correlated with higher dissolved oxygen levels in a single stream (Stone et al. 2004). A high water temperature sustained at > 25 °C can result in *M. falcata* extirpation (Rodland et al. 2009; Stagliano 2023).

Native freshwater mussels are also considered to be a “first food” by multiple Columbia plateau tribes in the Pacific Northwest (Quaempts et al. 2018). Indigenous groups like the Confederated Tribes of the Umatilla Indian Reservation (CTUIR) have significantly contributed to decades of freshwater mussel research. In particular, the CTUIR Freshwater Mussel Project specifically aims to restore self-sustaining freshwater mussel populations and preserve existing populations (Maine and O’Brien 2025). However, while the ecological and cultural importance of native freshwater mussels are well established, the specific distribution and abundance of *G. angulata* and *M. falcata* have been infrequently studied in the context of ecological associations, particularly at broad geographic scales (Blevins et al. 2017). Invasive freshwater bivalves have been investigated in the CRB (Hassett et al. 2017; Bolam et al. 2019; Henricksen and Bollens 2022; Robb‐Chavez et al. 2023), but not yet in the context of native freshwater mussel distribution, abundance, and/or ecological associations.

*Corbicula fluminea* (Müller, 1774), or the Asian clam, was introduced into the CRB in the 1930s as a possible food source for humans or with ballast water and has since colonized much of the river system (McMahon 2002; Dexter et al. 2015; Hassett et al. 2017; Dexter et al. 2020; Henricksen and Bollens 2022; Robb‐Chavez et al. 2023). Ecological variables associated with *C. fluminea* abundance include positive correlations with higher dissolved oxygen concentrations (Pereira et al. 2017; Henricksen and Bollens 2022), higher water temperature (Mincy 2019; Robb‐Chavez et al. 2023), neutral to basic pH levels (McDowell et al. 2014), and finer substrate (Sousa et al. 2008; Robb‐Chavez et al. 2023). Successful dispersal and broad distribution are likely related to generalist habitat preferences and effective veliger (larval) dispersal (McMahon 2002). In comparison to glochidia (the parasitic larval phase of native freshwater mussels), *C. fluminea* veligers are free-floating, planktonic larvae (McMahon 2002). Produced asexually via androgenesis (Hedtke et al. 2008; Pigneur et al. 2012), the planktonic nature of these veligers results in effective primary dispersal via flow, and secondary dispersal via domestic trade and recreation (Karatayev et al. 2007; Lucy et al. 2017).

The dispersal success and rapid population growth of *C. fluminea* in freshwater aquatic systems worldwide have likely had negative effects on native freshwater mussels (Ferreira-Rodríguez et al. 2019). *Corbicula fluminea* is characterized by swift growth rates, early maturation, and high fecundity that allow it to compete with native mussels for benthic space and nutrient resources (Strayer 1999; Sousa et al. 2008), which can limit native mussel growth (Haag et al. 2021). Dense populations of *C. fluminea* can be more vulnerable to die-offs due to high water temperatures and low dissolved oxygen levels (Cherry et al. 2005), and resultant ammonia production could negatively affect proximate native mussel populations. High filtration and excretion rates of *C. fluminea* are negatively correlated with the survival of native mussel glochidia, likely due to direct mortality and high ammonia production (Modesto et al. 2019). Widespread distribution and high abundance of invasive *C. fluminea* are potentially detrimental to *G. angulata* and *M. falcata* in North America. However, Kelley et al. (2022) identified positive associations between native mussel biomass and the presence and density of *C. fluminea*, indicating possible positive interactions such as native mussel facilitation of *C. fluminea* invasions. Evaluation of the current distribution and abundance of freshwater bivalves in relation to environmental variables can distinguish species-specific ecological associations and provide insight into effective management strategies which include conservation of native bivalve populations and mitigation of invasive bivalve expansion.

This aims of this study were to better elucidate the ecological associations between and among freshwater bivalves of the CRB and investigate linkages to bivalve distribution and abundance. The specific objectives were twofold. First, determine the distribution and abundance of *G. angulata*, *M. falcata*, and *C. fluminea* in the CRB; and second, quantify ecological associations of *G. angulata*, *M. falcata*, and *C. fluminea* in the CRB. We employed widespread snorkel surveys and environmental data collection, followed by geospatial data collection, model set construction, and model selection. The comparison of ecological associations and random effects quantified their respective explanatory value in relation to bivalve distribution and abundance. Identification of key habitat preferences and environmental covariates of bivalve distribution and abundance can inform species-specific management strategies in the CRB and elsewhere. This is particularly relevant to native mussels of varied risk statuses and an invasive clam with an expanded distribution.

## Methods

### Study sites

**Fig. 1:**
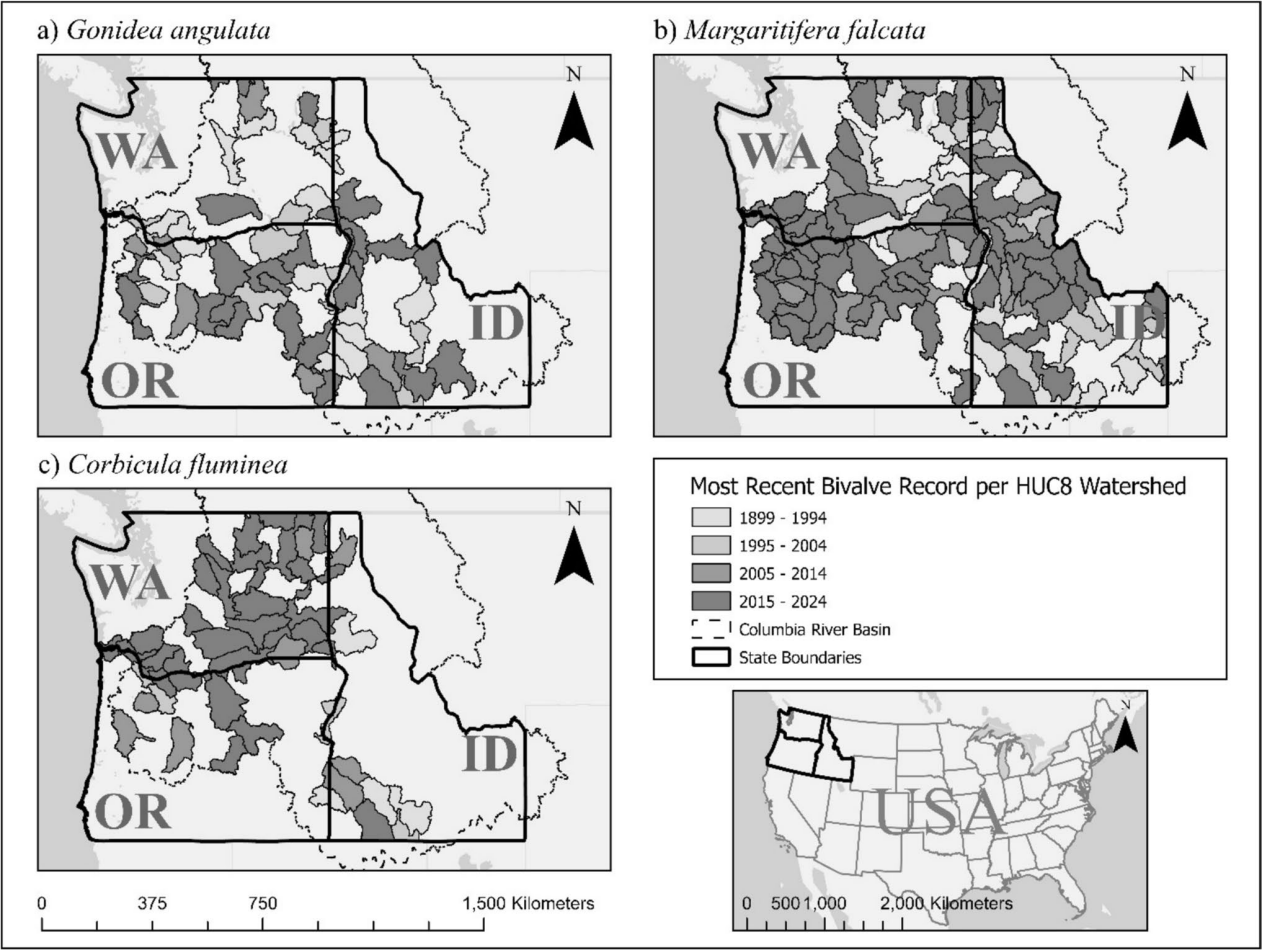
The most recent historical freshwater bivalve observation records for **a** *Gonidea angulata* (western ridged mussel), **b** *Margaritifera falcata* (western pearlshell), and **c** *Corbicula fluminea* (Asian clam) per hydrologic unit code (*HUC8*) watershed in Idaho, Oregon, and Washington within the boundary of the Columbia River Basin (CRB). Watersheds with more recent observation records are* shaded darker* compared to older records. Watersheds with no historical records for a species are not delineated or shaded

The study sites were selected based on historical native freshwater mussel presence records provided by the CTUIR and the Xerces Society for Invertebrate Conservation. Due to the sparse distribution of native freshwater mussels, we chose to focus on watersheds in the CRB for which there are prior records (Fig. 1) that are publicly accessible. Narrowing down the number of sites to those with records of either of these two native mussel species also made it possible to accommodate a small field crew, with a single snorkel surveyor, and the short (summer) field seasons necessitated by high spring and autumn flows and wildfire prevalence. At each study site, from one to five 50-m survey reaches were designated and surveyed, based on safety considerations and accessibility of the river. All visual bivalve identification, abundance counts, and host fish presence assessments were conducted by the lead author, N. G. Neal, to obviate inter-observer variability.

### Field data collection

**Fig. 2:**
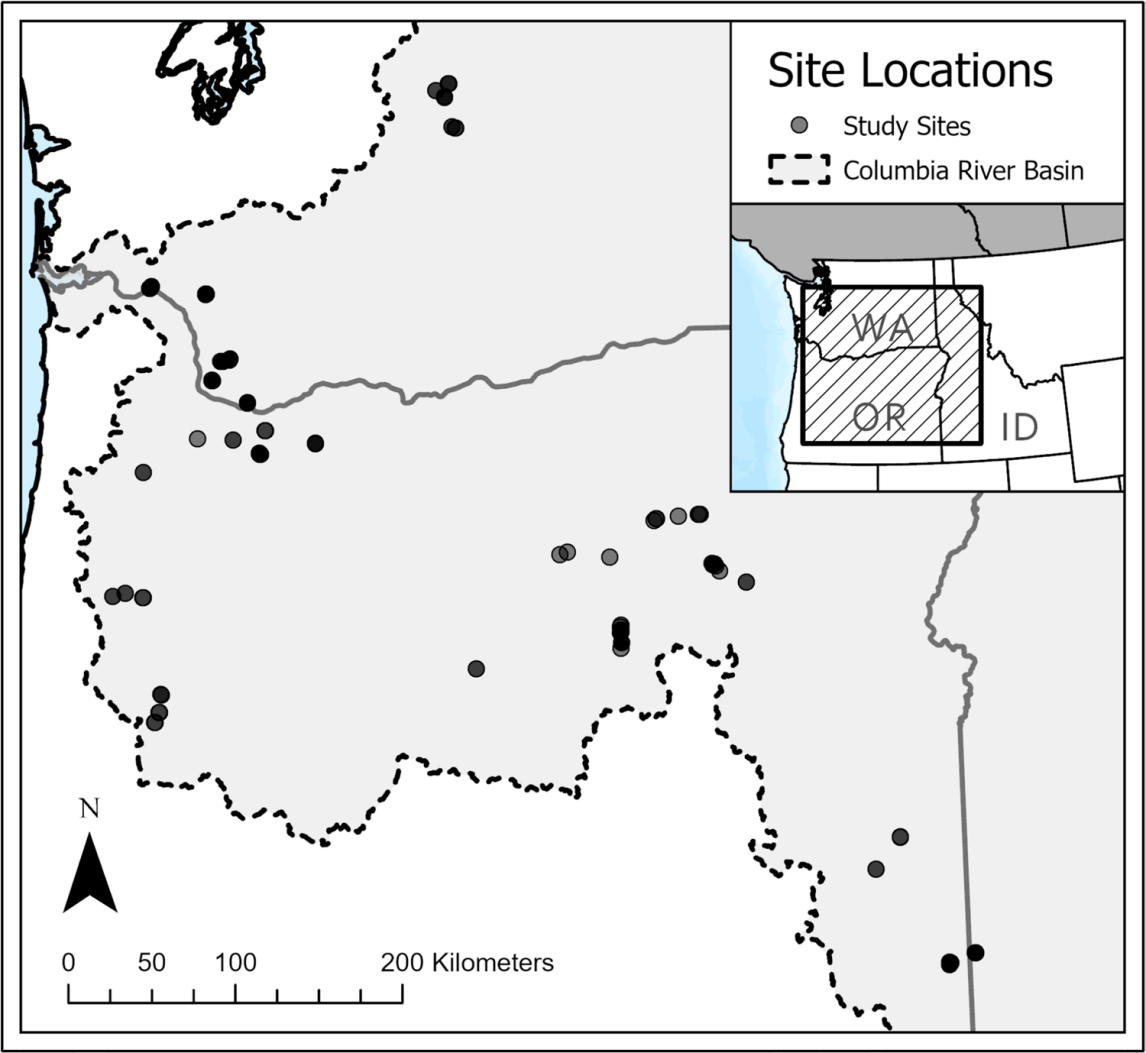
Locations of all 49 sites (containing one hundred and forty-seven 50-m survey reaches) are indicated by the* black circles*. The CRB is outlined to indicate the region of interest for this study

We performed snorkel surveys in the summers of 2023 and 2024 to count and identify bivalves and measure abiotic and biotic environmental variables at each 50-m survey reach (Fig. 2). Snorkel surveys were designed as one-person-hour visual assessments of the benthos of any given survey reach to record live abundance counts of *G. angulata*, *M. falcata*, and *C. fluminea* on a dive slate. Host fish (sculpin and salmonids) were identified during the snorkel survey and their presence was recorded on a dive slate; specific identifications were aided by the use of a field guide (Page and Burr 2011). The snorkel survey study design was based on the Visual Survey Protocol Framework for Western North American Freshwater Mussels (Blevins et al. 2024). In summary, these snorkel surveys consisted of (1) the designation of a 50-m section of the river, followed by (2) the measurement of environmental variables (described below), and (3) a one-person-hour visual snorkel survey with an unobstructed view of the river bottom and without excavation of substrate. This method is recommended for low mussel abundance (density) and the confirmation of mussel presence in conjunction with the collection of data on environmental covariates due to time and spatial efficiency (1 person-hour per 50 m) (Blevins et al. 2024). Fieldwork permits were not necessary due to the non-disruptive nature of these surveys and the site locations on public-access land.

**Fig. 3:**
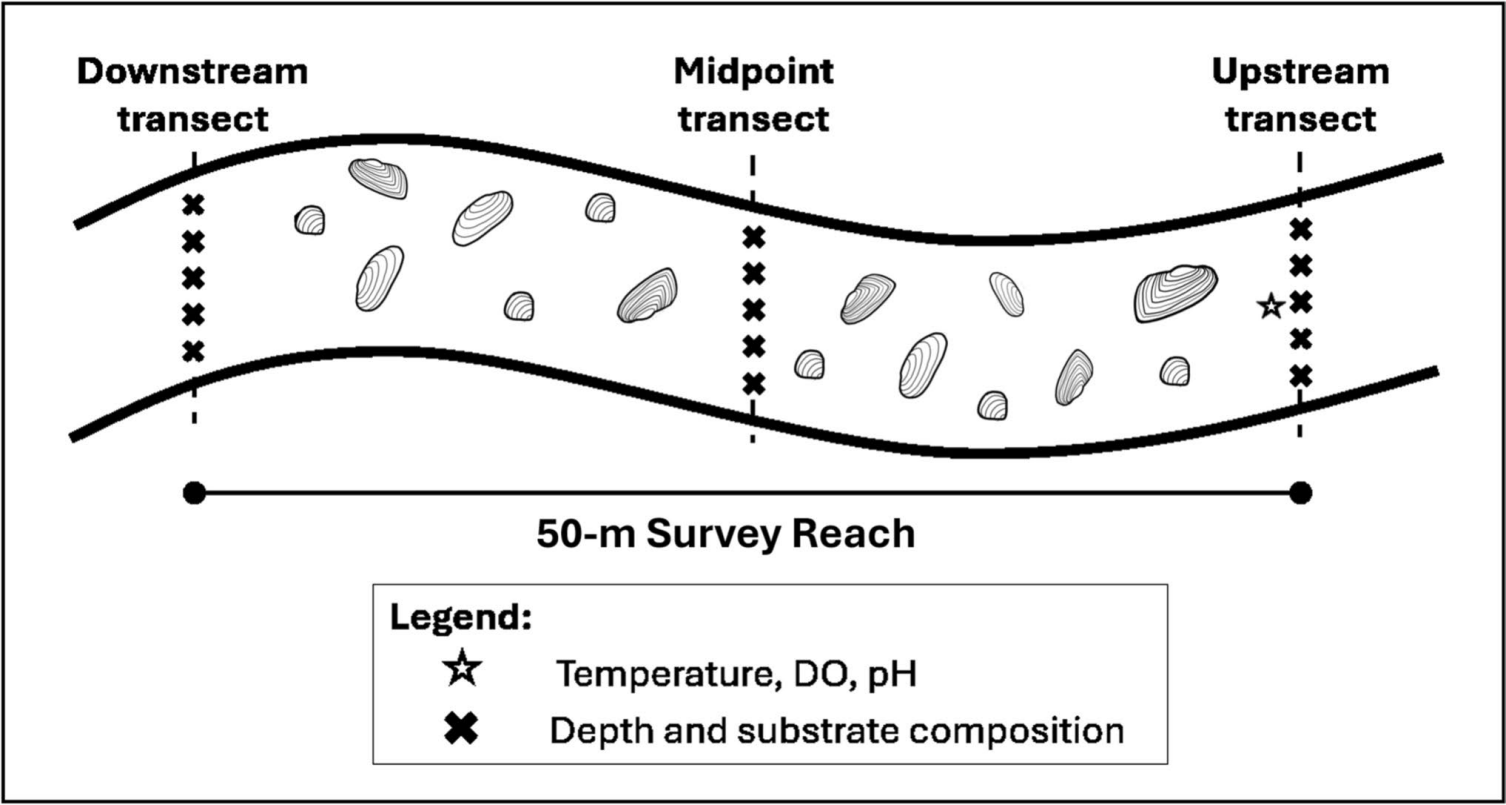
Sampling design for each 50-m survey reach. Depth and substrate measurements were collected at equidistant points across downstream, midpoint, and upstream transects. Point measurements of water temperature, dissolved oxygen, and pH were collected at the midpoint of the upstream transect

Water quality and geomorphology variables were measured at each survey reach, at the specific locations shown in Fig. 3. Water temperature (°C), dissolved oxygen concentration (mg L^−1^), and pH were recorded with an optical dissolved oxygen meter (Apera DO 850; Apera Instruments, Columbus, OH) and pH meter (Hach Pocket Pro; Hach, Loveland, CO), respectively, at 10 cm below the water surface at the mid-channel point of the most upstream cross-channel transect. In addition, ten equidistant substrate samples were collected perpendicular to river flow and measured with a gravelometer cobble scale (Wildco, Yulee, FL) along three cross-channel transects: one immediately upstream, one at the midpoint, and one immediately downstream of each 50-m survey reach. The measured substrate samples were then used to calculate the mean substrate size (MEANSUB), the proportion of sand among the samples (PROPSAND), and the proportion of boulder among the samples (PROPBLDR). Water depth (cm) was measured at five equidistant points across the same three upstream, midpoint, and downstream cross-channel transects of each survey reach. Pools and low-velocity flow were identified in each reach via visual assessment and their proportion of the total reach length was recorded (PROPPOOL).

### Geospatial data collection and analyses

All geospatial analyses were conducted in ArcGIS Pro (Esri 2024). We incorporated five open-access geospatial and climate data variables for our analyses (Table 1).

**Table 1:** All open-access geospatial and climate data variables included in the analyses, accompanied by the respective resolution, raster layer, and source

Variable	Resolution	Raster layer	Source
Mean annual temperature 1991–2020 (°C) (MEANTEMP)	800 m	PRISM 30-year normals	PRISM Climate Group (2024)
Minimum annual temperature 1991–2020 (°C) (MINTEMP)	800 m	PRISM 30-year normals	PRISM Climate Group (2024)
Mean annual temperature 1991–2020 (°C) (MAXTEMP)	800 m	PRISM 30-year normals	PRISM Climate Group (2024)
Elevation (m)	10 m	US Geological Survey national map	US Geological Survey (2024)
Percent impervious surface (%) (IMPSURF)	30 m	National Land Cover Database 2021	Dewitz (2023)

We calculated the 1991–2020 climate normals [mean annual air temperature 1991–2020 (MEANTEMP), maximum annual air temperature 1991–2020 (MAXTEMP), and minimum annual air temperature 1991–2020 (MINTEMP)] and elevation for each reach as the mean of the upstream point and downstream point values of the reach. Percent impervious surface (IMPSURF) was calculated per 1-km buffer for each survey reach. Mean annual precipitation was not included in our final suite of models due to high collinearity with elevation, minimum annual air temperature, and maximum annual air temperature.

### Statistical analyses

All data and statistical analyses were conducted with R (version 4.3.2; R Core Team 2023) and performed separately for each of the three bivalve species of interest. We assessed the presence/absence as well as the abundance of each bivalve species per 50-m survey reach in relation to environmental variables. We log-transformed PROPSAND, PROPBLDR, IMPSURF, and PROPPOOL due to the non-normal nature of these variables and evaluated for collinearity among the variables using the corrplot function (Wei and Simko 2021). To assess the explanatory value of the environmental variables, we then constructed a model set comprising a null model, univariate models, and multivariate models (Appendix A) based on an information-theoretic approach (Burnham et al. 2011) and using a collinearity cutoff value of *r* = 0.7.

To evaluate bivalve presence and absence in relation to environmental variables we employed binomial generalized linear mixed-effects models with hydrologic unit code 8 delineation of watersheds (hereafter “watershed”) as a random effect. To evaluate bivalve abundance in relation to environmental variables, we constructed Poisson generalized linear mixed-effects models with watershed as a random effect. We included watershed as a random effect to account for different numbers of sites per watershed, and surveyed reaches per site, as addressed in the “Study sites” section above. The model sets were evaluated and ranked via Akaike information criterion adjusted for small sample size (AICc) with the aictab function in the AICcmodavg R package (Mazerolle 2020). We assessed the normality of scaled residuals via a simulation-based approach with the DHARMa package (Hartig  2024). To determine the relative strength of fixed and random effects, we calculated marginal and conditional Nakagawa pseudo* R*^2^ values using the r2_nakagawa function (Lüdecke et al. 2021). Lastly, we calculated regression coefficients (β) and Wald’s 95% confidence intervals (R Core Team 2024).

## Results

### Environmental variables

Over two field seasons (summer 2023 and summer 2024), we visited 49 study sites and surveyed a total of one hundred and forty-seven 50-m reaches, of which 82 were in Oregon, 60 in Washington, and five in Idaho (Fig. 2). Water quality varied considerably across the 147 surveyed reaches: pH ranged from 7.0 to 8.9, dissolved oxygen concentrations from 5.7 mg L^−1^ to 11.6 mg L^−1^, and water temperature from 9.3 °C to 27.9 °C (these measurements did not account for diel or seasonal variation). Higher pH levels, lower dissolved oxygen concentrations, and higher water temperatures were more frequently recorded at survey reaches in eastern Oregon and Idaho. Geomorphology and PROPPOOL were also variable: MEANSUB ranged from 17.8 mm to 188.8 mm, PROPSAND ranged from 0 to 0.70, PROPBLDR ranged from 0 to 0.43, mean depth ranged from 14.9 cm to 139.2 cm, and PROPPOOL ranged from 0 to 1. These microhabitat variables did not vary spatially in any obvious pattern. In terms of geospatial and climate variables, mean elevation ranged from 4.6 m to 1454.6 m above sea level, MEANTEMP ranged from 5.4 °C to 11.9 °C, MAXTEMP ranged from 23.2 °C to 32.8 °C, MINTEMP ranged from −9.4 °C to 1.4 °C, and IMPSURF ranged from 0.0011 to 0.39. Higher elevations, higher MEANTEMP, higher MINTEMP, higher MAXTEMP, and lower IMPSURF were all more frequently observed east of the Cascade Mountain Range.

### Distribution and abundance of the native mussels* Gonidea angulata* and* Margaritifera falcata*, and the invasive clam* Corbicula fluminea*

**Fig. 4:**
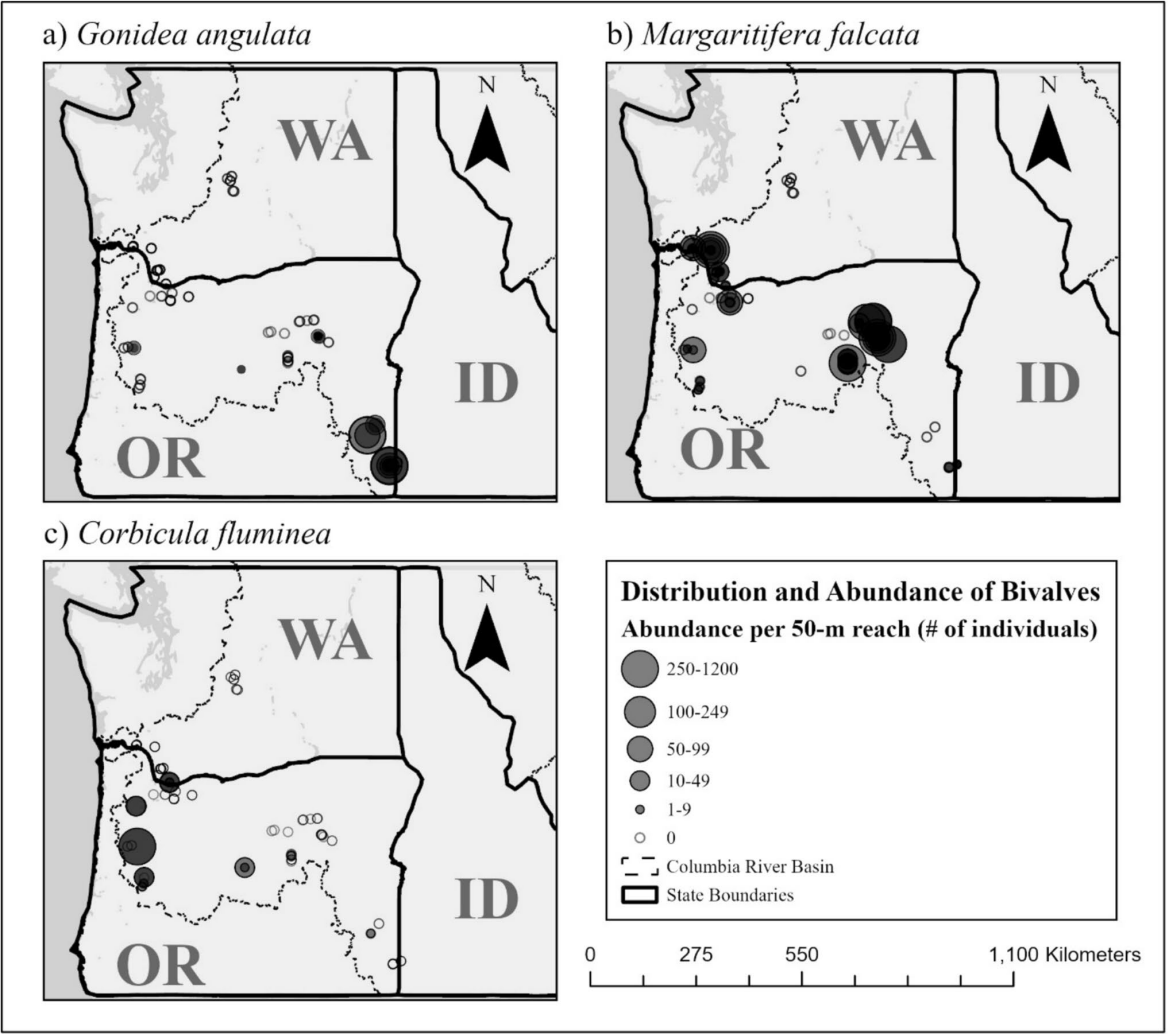
The abundance of **a** *Gonidea angulata*, **b** *Margaritifera falcata*, and **c** *Corbicula fluminea* at each survey reach, with the number of individuals illustrated by graduated* filled circles*. Survey reaches where each of these species is absent are delineated by* open circles*

We identified native and invasive freshwater bivalves at survey reaches across the CRB (Fig. 4). The occurrence of the native mussel *G. angulata* was largely limited to eastern Oregon and the rural Owyhee River Basin (Fig. 4a). We identified 2330 live *G. angulata* across 22 survey reaches, with a range in abundance from 1 to 725 per 50-m survey reach. In contrast, we observed *M. falcata* to occur at a higher number of survey reaches (76) distributed widely across Oregon and southwest Washington (Fig. 4b). In total, we observed 6703 live *M. falcata*, with abundance ranging from 1 to 1140 per 50-m survey reach. Among the native mussels (*G. angulata* and *M.* falcata), we observed mainly adults, with juvenile mussels identified at fewer than ten sites. The presence of *C. fluminea* was widespread geographically, but it occurred at only 16 survey reaches (Fig. 4c), with 793 live individual *C. fluminea* observed, ranging in abundance from 1 to 300 per 50-m survey reach.

### Ecological associations of the native mussel* Gonidea angulata*

**Fig. 5a, b:**
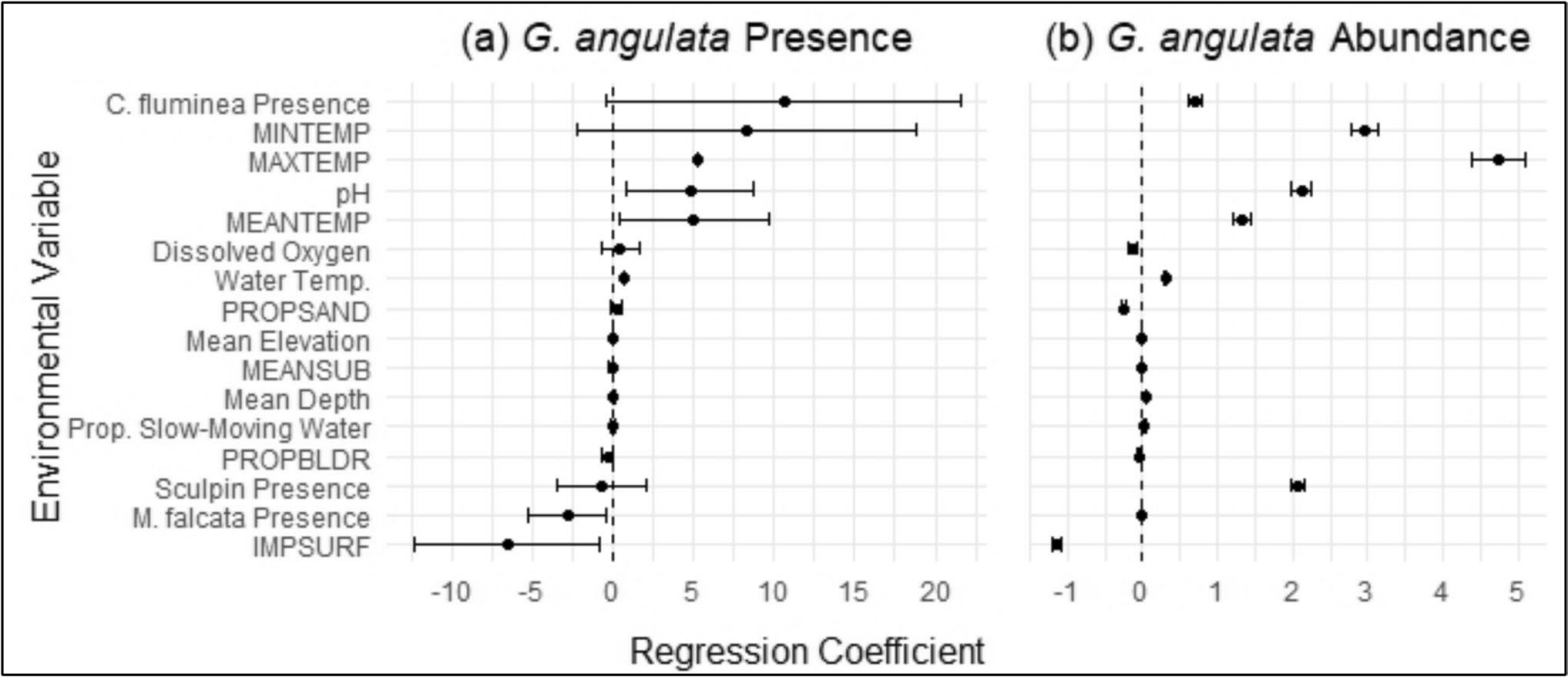
Regression coefficients for all 16 environmental variables are plotted for *Gonidea angulata* presence and abundance with Wald’s 95% confidence intervals. **a** *Gonidea angulata* presence assessed via binomial generalized linear mixed models (GLMMs), and **b** *G. angulata* abundance assessed via Poisson GLMMs

Variation in presence and/or abundance of *G. angulata* was best explained by microhabitat variables (e.g., water temperature) and landscape variables (e.g., IMPSURF, MEANTEMP, and MAXTEMP). All statistical summaries for the top models (as ranked by AICc selection) for *G. angulata* are provided in Table 2 with the standardized effect sizes illustrated in Fig. 5. We found that the environmental variable most strongly associated with the presence or absence of *G. angulata* was IMPSURF. The top binomial generalized linear mixed model (GLMM) was a univariate model of IMPSURF. The next two best models, both within two degrees of AICc separation, were a multivariate model of IMPSURF and MEANTEMP, and a multivariate model of IMPSURF and water temperature. Inclusion of watershed as a random effect in each model consistently increased explanatory value, as evidenced by the difference between marginal* R*^2^ and conditional* R*^2^ values. In evaluating environmental variables associated with the total abundance of *G. angulata*, a univariate Poisson GLMM model showed that the strongest association was a positive relationship with MAXTEMP. However, we determined that the scaled residuals of the Poisson GLMMs were highly overdispersed; this remained consistent with negative binomial distribution and zero-inflated models.

**Table 2:** Statistical summaries for the top models for *Gonidea angulata* (western ridged mussel) presence (binomial) and abundance (Poisson)

Model type	Model	Marginal* R*^2^	Conditional* R*^2^	β (95% CI)	*p*-value
Presence (binomial)	IMPSURF	0.150	0.995	− 6.58 (− 12.3, − 0.88)	0.024*
	IMPSURF + MEANTEMP	0.067	0.996	− 6.74 (− 13.9, 0.371)	0.063
				3.05 (− 3.10, 9.20)	0.331
	IMPSURF + Water temperature	0.165	0.994	− 6.29 (− 12.1, − 0.491)	0.034*
				0.273 (−0.603, 1.15)	0.610
Abundance (Poisson)	MAXTEMP	0.519	0.999	4.74 (4.39, 5.09)	< 0.001***

*β* Regression coefficient,* CI* confidence interval; for other abbreviations, see Table 1

* *p* < 0.05, *** p < 0.001

### Ecological associations of the native mussel* Margaritifera falcata*

**Fig. 6a, b:**
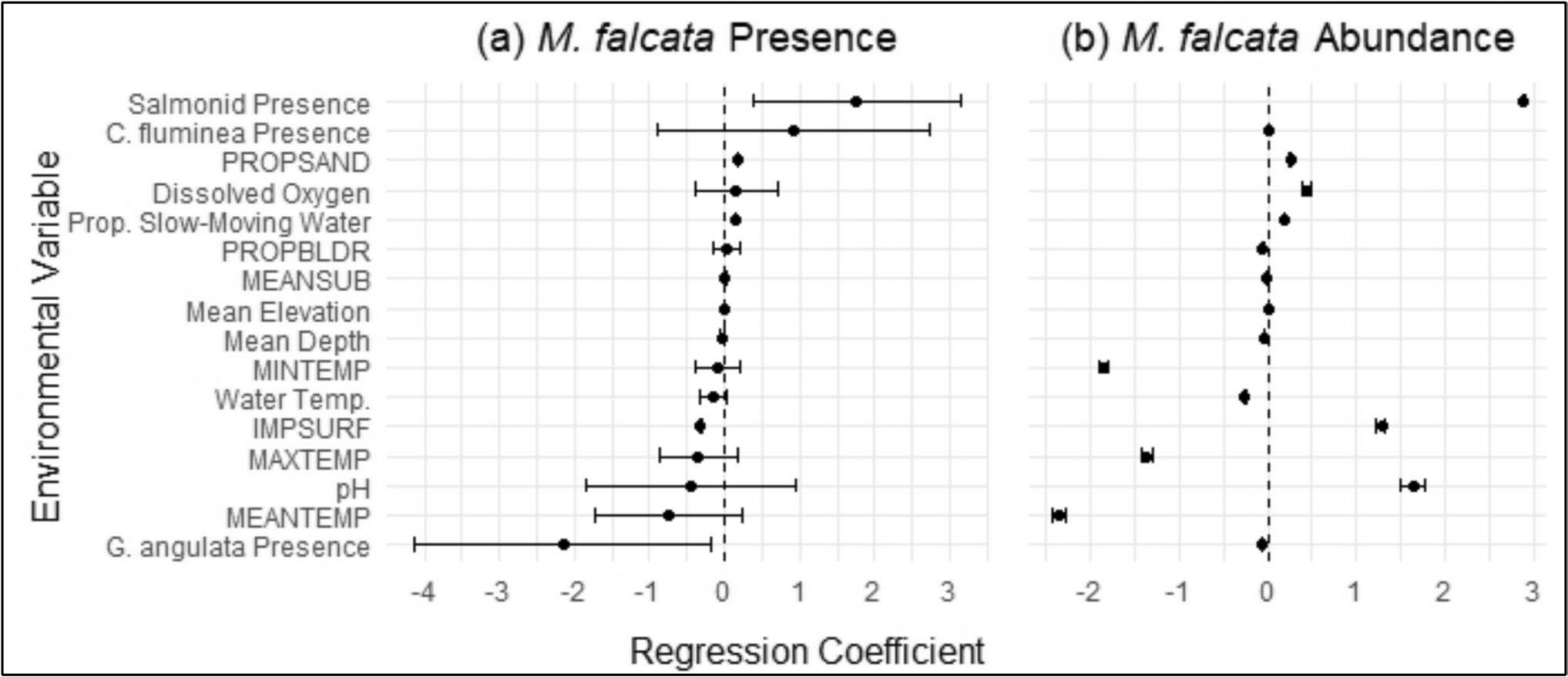
Regression coefficients for all 16 environmental variables are plotted for *Margaritifera falcata* presence and abundance with Wald’s 95% confidence intervals. **a** *Margaritifera falcata* presence assessed via binomial GLMMs, and **b** *M. falcata* abundance assessed via Poisson GLMMs

Variation in *M. falcata* presence and/or abundance was best explained by biotic variables (e.g., presence of salmonids, *G. angulata*, and *C. fluminea*), as well as microhabitat variables (e.g., dissolved oxygen and water temperature). All statistical summaries for the top models (as ranked by AICc selection) for *M. falcata* are provided in Table 3 with the standardized effect sizes illustrated in Fig. 6*.* The top binomial GLMM explaining variation in *M. falcata* presence and absence was a multivariate model of salmonid presence, *G. angulata* presence, and *C. fluminea* presence. This top model was followed by a univariate model of salmonid presence, then a multivariate model of *C. fluminea* presence and *G. angulata* presence. Inclusion of watershed as a random effect in each model consistently increased the explanatory value, as evidenced by the difference between marginal* R*^2^ and conditional* R*^2^ values. To evaluate variation in *M. falcata* abundance, the top Poisson GLMM was a multivariate model of dissolved oxygen and elevation. However, we again determined that the scaled residuals of the Poisson GLMMs were highly overdispersed; this remained consistent with negative binomial distribution and zero-inflated models.

**Table 3:** Statistical summaries for the top models for *Margaritifera falcata* (western pearlshell) presence (binomial) and abundance (Poisson)

Model type	Model	Marginal* R*^2^	Conditional* R*^2^	β (95% CI)	*p*-value
Presence (binomial)	Salmonid presence + *Corbicula fluminea* presence + *Gonidea angulata* presence	0.118	0.741	1.42 (− 0.08, 2.93)	0.064
				1.81 (− 0.47, 4.09)	0.119
				− 2.32 (− 4.75, 0.11)	0.061
	Salmonid presence	0.061	0.639	1.76 (0.38, 3.15)	0.013*
	*C. fluminea* presence + *G. angulata* presence	0.098	0.745	1.96 (− 0.35, 4.29)	0.098
				− 2.91 (− 5.28, − 0.54)	0.016*
Abundance (Poisson)	Dissolved oxygen + Elevation	0.372	0.999	0.098 (0.046, 0.018)	< 0.001***
				0.018 (0.017, 0.018)	< 0.001***

For abbreviations, see Table 2

* *p* < 0.05, *** p < 0.001

### Ecological associations of the invasive clam* Corbicula fluminea*

**Fig. 7a, b:**
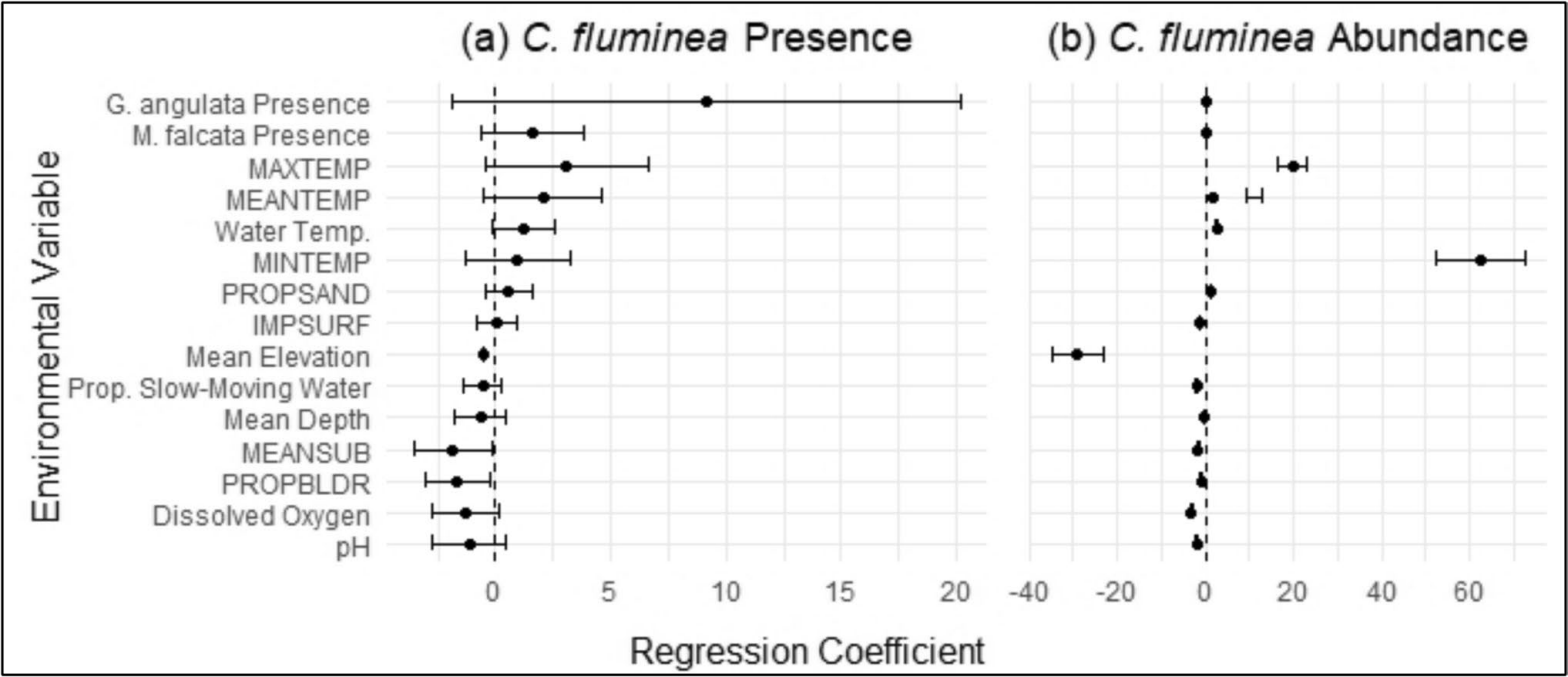
Regression coefficients for all 15 environmental variables are plotted for *Corbicula fluminea* presence and abundance with Wald’s 95% confidence intervals. **a** *Corbicula fluminea* presence assessed via binomial GLMMs, and **b** *C. fluminea* abundance assessed via Poisson GLMMs

Variation in the presence and/or abundance of *C. fluminea* was best explained by the presence of *G. angulata,* microhabitat variables (e.g., PROPBLDR and MEANSUB) and landscape-scale variables (e.g., MEANTEMP and IMPSURF). All statistical summaries for the top models (as ranked by AICc selection) for *C. fluminea* are listed in Table 4 with the standardized effect sizes illustrated in Fig. 7. Evaluation of *C. fluminea* presence and absence revealed the top binomial GLMM to be a univariate model of *G. angulata* presence. Other top models included a univariate model of MEANSUB, and a multivariate model of *G. angulata* presence and *M. falcata* presence. Inclusion of watershed as a random effect in each model consistently increased explanatory value, as evidenced by the difference between marginal* R*^2^ and conditional* R*^2^ values**.** In evaluating the variation in *C. fluminea* abundance via Poisson GLMMs, the top model was a multivariate model of MEANTEMP and IMPSURF. However, we again determined that the scaled residuals of the Poisson GLMMs were highly overdispersed; this remained consistent with negative binomial distribution and zero-inflated models.

**Table 4:** Statistical summaries for the top models for *Corbicula fluminea* (Asian clam) presence (binomial) and abundance (Poisson)

Model type	Model	Marginal* R*^2^	Conditional* R*^2^	β (95% CI)	*p*-value
Presence (binomial)	*Gonidea angulata* presence	0.151	0.954	9.15 (− 1.90, 20.19)	0.105
	MEANSUB	0.031	0.970	− 0.05 (− 0.09, − 0.01)	0.028*
	*G. angulata* presence + *Margaritifera falcata* presence	0.152	0.958	9.52 (− 2.42, 21.45)	0.118
				1.48 (− 0.98, 3.94)	0.239
Abundance (Poisson)	MEANTEMP + IMPSURF	0.040	0.999	0.28 (0.21, 0.35)	< 0.001***
				− 1.03 (− 1.14, -0.92)	< 0.001***

For abbreviations, see Tables 1 and 2

* *p* < 0.05, *** p < 0.001

## Discussion

We observed high variability in distribution and abundance among all three species of freshwater bivalves studied in the CRB. *Gonidea angulata*, the most threatened of the two native mussel species in our study (Blevins et al. 2017), was very limited in its distribution and largely confined to rural areas rather than highly developed or urbanized regions of the CRB (Fig. 4a). An example of this was its frequent occurrence and high abundance within non-agricultural regions of the Owyhee Canyonlands in southeastern Oregon. The distribution of *G. angulata* has been poorly described in the USA compared to the more substantial documentation of the Canadian range of this species (Stanton et al. 2012; Snook et al. 2019; Wade et al. 2025). However, range reduction of *G. angulata* has been documented in the CRB (Blevins et al. 2017), with evidence of mass die-off events at multiple sites of historical abundance (see literature review in Blevins et al. 2020). The sparse distribution of *G. angulata* that we observed could therefore have resulted from both historical range reduction and the frequency of mass die-off events.

*Margaritifera falcata*, the other native mussel species included in our study, was observed at the highest number of survey reaches amongst the three bivalves studied and was broadly distributed across the CRB (Fig. 4b). Compared to *G. angulata*, *M. falcata* has been more commonly observed and has a well-defined distribution, with historical records indicating a range from southern California to central British Columbia, and east to Colorado (Blevins et al. 2017; Scully-Engelmeyer et al. 2023). Therefore, it was unsurprising to observe *M. falcata* across a wider distribution compared to *G. angulata*.

We observed the invasive clam *C. fluminea* in relatively fewer survey reaches, although it was widely distributed across the CRB, including regions of large-scale human development (Fig. 4c). For instance, *C. fluminea* has commonly been observed in populous and heavily recreated tributaries of the Willamette River, Oregon (Baker et al. 2004) and in the lower Columbia River (Dexter et al. 2020; Hassett et al. 2017; Henricksen and Bollens 2022; Robb‐Chavez et al. 2023). Primary dispersal of *C. fluminea* is attributed to ballast water and international trade, while secondary dispersal can be facilitated by domestic trade, recreational fishing, and fish stocking (Karatayev et al. 2007; Lucy et al. 2017). A combination of these dispersal mechanisms may contribute to its distribution near areas of anthropogenic disturbance and its widespread dispersal across the CRB (Hassett et al. 2017). We found shells of *C. fluminea* at a much higher number of survey reaches than live individuals, potentially due to the vulnerability of dense populations to die-off due to high temperature and low dissolved oxygen conditions (Cherry et al. 2005).

### Ecological associations with bivalve presence

The presence of co-occurring freshwater bivalve species emerged as an important variable associated with the presence of *M. falcata* and may be related to either interspecific effects between bivalves or, alternatively, overlap in their ecological associations. *Margaritifera falcata* presence was significantly negatively correlated with the presence of the native *G. angulata*. There were no environmental variables shared among the top models to explain *G. angulata* and *M. falcata* presence or abundance. Historical native ranges of *M. falcata* and *G. angulata* overlapped across much of the CRB, but population declines and range reductions have reduced the current range overlap (Blevins et al. 2017). Future research on the feeding dynamics of *G. angulata* and *M. falcata*, similar to what has been undertaken for *C. fluminea* feeding intensity and preferences (Bolam et al. 2019), could elucidate possible competition for food between different species of native mussels in the CRB.

Stable substrate and flow refuges are frequently stated as habitat preferences of freshwater mussels, often attributed to the presence of boulders in riverine reaches (Vannote and Minshall 1982; Strayer 1993; Brown et al. 2010; Hegeman et al. 2014). There is also a documented native freshwater mussel preference for small-grained substrate (e.g., sand) in interstitial spaces between large-grained substrate (e.g., boulder) (Vannote and Minshall 1982; Stanton et al. 2012; Davis et al. 2013; Snook et al. 2019). However, the only substrate composition metric to emerge in our top models was a significant, negative correlation between MEANSUB and *C. fluminea* presence. This is consistent with the documented preference of *C. fluminea* for small substrate composition and high embeddedness, with its ability to persist in habitats prone to disturbance (Sousa et al. 2008; Robb‐Chavez et al. 2023). Although substrate is frequently cited as an important habitat preference of native mussels, the wide variation in observed substrate composition and the strong random effect of watershed differences may contribute to the lack of strong association found in our study.

The proportion of impervious surface (IMPSURF) appeared as a variable in all three top models explaining variation in *G. angulata* presence, with a strong negative association. *Gonidea angulata* was the only species with IMPSURF as a top predictor of presence, and it frequently occurred in rural areas of eastern Oregon. An assessment of IMPSURF can be used to describe rocky or mountainous habitat, but it also serves as a proxy variable for the degree of anthropogenic disturbance (Sutton et al. 2009) by accounting for roads, buildings, parking lots, and other anthropogenic developments. It may be related to frequent recreation or prior construction activity, both of which are potential threats to native mussel beds. In addition to direct habitat destruction, the presence of proximate impervious surfaces can lead to an increase in runoff and sedimentation, limiting habitat suitability for freshwater mussels (Bukaveckas et al. 2023). Freshwater mussels at the benthic, slow-moving adult stage may be particularly susceptible to anthropogenic impacts as they are dispersal limited and have a reduced ability to escape direct disturbance events.

Inclusion of watershed as a random effect was of considerable importance across all of our models for all three bivalve species. Like the high variation in bivalve distribution and abundance, the strength of this random effect could be attributed in part to the limited dispersal capabilities of freshwater bivalves as benthic, slow-moving organisms reliant on host fish for dispersal (O’Brien et al. 2013). Such variation in distribution may be exacerbated by aquatic habitat fragmentation. More thoroughly studied in salmonids (Sheer and Steel 2006), the loss of aquatic habitat connectivity can in turn affect mussel dispersal by reducing host fish accessibility. Sculpin, host fish for *G. angulata,* are largely sedentary as adults, with minimal upstream or downstream migration (Petty and Grossman 2004; Radinger and Wolter 2014). Reliance on sculpin hosts will allow mussel glochidia to disperse sufficiently to develop new mussel beds within a small geographic area, but likely does not result in frequent inter-watershed movement across larger spatial scales. Salmonids, the host fish for *M. falcata,* are far more widespread, but are under threat, e.g., populations in the CRB are limited by fewer anadromous fish returns and range encroachment by invasive fish (Tremblay et al. 2016). The importance of salmonids to *M. falcata* is supported by the finding in both of our GLMM models that salmonid presence was positively correlated with the presence of *M. falcata.*

### Ecological associations with bivalve abundance

The abundance data for all three bivalves were all strongly overdispersed, as were the scaled residuals for all top Poisson GLMMs, and thus these findings need to be interpreted with caution. Nevertheless, we have included the top bivalve abundance models in our results above and we discuss them briefly here.

The abundance of the native mussel *G. angulata* was significantly positively correlated with MAXTEMP. *Gonidea angulata* and their host fish sculpin are both documented to have high acute and chronic thermal tolerances (Valeria et al. 2025) and thus better resistance to high temperature conditions. We observed a positive association of *M. falcata* abundance with high dissolved oxygen concentration, consistent with other studies of native mussels (Chen et al. 2001; Stone et al. 2004). High sedimentation and silt composition can reduce dissolved oxygen concentrations and negatively affect the ability of native freshwater mussels to obtain oxygen (Stone et al. 2004; Snook et al. 2019). Elevation was also positively correlated with *M. falcata* abundance and appeared in the top model, but had an extremely small regression coefficient.

*Corbicula fluminea* abundance was positively correlated with MEANTEMP. Warming air and water temperatures are positively correlated with increased dispersal of aquatic invasive species such as *C. fluminea* (Mincy 2019; Robb‐Chavez et al. 2023) and can accelerate the production of veliger drift mechanisms (mucocytes) and stimulate veliger flotation (Rosa et al. 2012). Unexpectedly, *C. fluminea* abundance was negatively correlated with IMPSURF, although it is well-documented that this species relies on secondary dispersal via anthropogenic activities (Karatayev et al. 2007; Lucy et al. 2017). Warming air and water temperatures in the CRB associated with a changing climate (Dexter et al. 2020) could accentuate the dispersal and population growth of this invasive clam. Although we did not observe direct negative effects of *C. fluminea* on native mussels, the continued range expansion and population increases of this invasive species could negatively affect freshwater mussels via potential competition for resources.

### Implications for management of native and invasive freshwater bivalves

Our findings have important implications for the conservation of native freshwater mussels and the management of invasive bivalves in the CRB and elsewhere. The wide variation in ecological associations that we observed, along with the strength of broad geographic (watershed) random effects, indicate that management for freshwater bivalves should not only be species-specific, but should also consider differences between watersheds. This conclusion is highly relevant to the large geographic area encompassed by the CRB, which experiences dramatic variations in climate, water quality, and anthropogenic disturbance across the region (Matheussen et al. 2000). Consideration of the ecological associations and population distribution indicated by these results can directly inform ongoing propagation and reintroduction efforts, such as those conducted by the CTUIR Freshwater Mussel Project (Maine and O’Brien 2025).

Identification of mussel age classes is also important for management strategies to distinguish actively reproducing populations versus remnant populations of historical mussel beds. Future research quantifying mussel distribution by age class would provide insight into which populations are, or are not, successfully reproducing. The majority of the mussels we observed were adults, with young mussels identified at fewer than ten sites (this was anecdotally recorded in our survey notes but not included in our formal analyses). Juvenile mussels tend to burrow deeper into substrate compared to adult mussels (Neves and Widlak 1987; Yeager et al. 1994). Because we did not excavate the substrate in this study, this may have contributed to the low number of juvenile mussels that we observed. The undercounting of juvenile mussels may be exacerbated by their smaller sizes compared to adult mussels.

We found different ecological associations among the three bivalve species studied, which suggests the need for species-specific management approaches. For instance, the prevalence of IMPSURF in our top models for *G. angulata* as a strongly negative correlate indicates possible vulnerability of this species to disturbance. Particular attention to human development and construction near *G. angulata* beds may therefore be critical for the conservation and management of these populations. In contrast, variation in *M. falcata* presence was best explained by biotic variables, including host fish and *G. angulata* presence. Salmonid management is of great interest and concern in the CRB, and its linkage to native mussel populations should be an important additional consideration to ongoing management, stocking, and restoration practices. Future studies could implement our findings on ecological associations to construct habitat suitability maps for native mussel taxa across the CRB.

## Conclusions

Our field observations and resulting statistical models found various biotic associations as well as landscape and climate variables to predict the presence and/or abundance of three bivalve species. In addition, the explanatory value of the models was consistently increased by the inclusion of watershed as a random effect. Broadly speaking, our results demonstrate that the dispersal-limited nature of bivalves can contribute to strong geographic (watershed) variation, which can offset fine-scale ecological associations. With native mussels often being long-lived and limited in their dispersal ability, their present distribution in the CRB is potentially more limited to that of residual populations from prior mussel beds than contemporary dispersal. Assessment of the distribution, abundance, and ecological associations of native and invasive bivalve species has important management implications. Scientific understanding of similarities in ecological associations of *C. fluminea* and native freshwater mussel species, and overlaps in their distributions, can better inform management of potentially deleterious invasive species.

## Electronic supplementary material

Below is the link to the electronic supplementary material.

Supplementary file1 (CSV 43 KB)

## Data Availability

Data are provided in the supplementary materials. Locality data are only provided to water body level due to the sensitive nature of freshwater mussel beds, but can be made available upon request to the corresponding author.

## Appendix A: The full model set of null, univariate, and multivariate models constructed, tested, and compared

Null

- Intercept only

Univariate

- pH
- Dissolved oxygen
- Water temperature
- Mean substrate size (MEANSUB)
- Mean depth
- Proportion of sand (PROPSAND)
- Proportion of boulder (PROPBLDR)
- Bivalve 1 abundance
- Bivalve 2 abundance
- Mean elevation
- Proportion of impervious surface (IMPSURF)
- Proportion slow water
- 1991–2020 mean annual air temperature (MEANTEMP)
- 1991–2020 minimum annual air temperature (MINTEMP)
- 1991–2020 maximum annual air temperature (MAXTEMP)
- Host fish presence

Multivariate

- pH + dissolved oxygen + water temperature
  o To assess aspects of water quality frequently associated with fish health.
- pH + WATERTEMP
  o To assess aspects of water quality frequently associated with fish health.
- MEANTEMP + IMPSURF
  o To assess possible effects of increased surface temperatures.
- Mean elevation + water temperature
  o To assess possible effects of headwater versus lowland streams.
- Host fish presence + Bivalve 1 + Bivalve 2
  o To assess possible effects of biotic variables including host fish presence and other native or invasive freshwater bivalve species.
- Bivalve 1 + Bivalve 2
  o To assess possible effects of the presence of other native or invasive freshwater bivalve species.
- PROPSAND + PROPBLDR
  o To assess possible effects of substrate composition, particularly small-sized substrates stabilized by boulders.
- Water Temperature + IMPSURF
  o To assess possible compounding effects of temperature pollution and impervious surface.
- Dissolved oxygen + elevation
  o To assess possible effects of headwater versus lowland streams.
- Depth + PROPBLDR
  o To assess possible effects of habitat stability related to larger substrate and higher flow quantity.
- MINTEMP + PROPBLDR
  o To assess possible effects of snowmelt and substrate stability.

## Abbreviations

CRB: Columbia River Basin
CTUIR: Confederated Tribes of the Umatilla Indian Reservation
GLMM: Generalized linear mixed model
IMPSURF: Proportion of impervious surface
MAXTEMP: Maximum annual air temperature 1991–2020
MEANSUB: Mean substrate size
MEANTEMP: Mean annual air temperature 1991–2020
MINTEMP: Minimum annual air temperature 1991–2020
PROPBLDR: Proportion of boulder
PROPSAND: Proportion of sand
PROPPOOL: Proportion of reach length observed as pools or low-velocity flow
